# Study protocol: optimising newborn nutrition during and after neonatal therapeutic hypothermia in the United Kingdom: observational study of routinely collected data using propensity matching

**DOI:** 10.1136/bmjopen-2018-026739

**Published:** 2018-10-23

**Authors:** Cheryl Battersby, Nick Longford, Mehali Patel, Ella Selby, Shalini Ojha, Jon Dorling, Chris Gale

**Affiliations:** 1 Neonatal Data Analysis Unit, Department of Medicine, Section of Neonatal Medicine, Imperial College London, London, UK; 2 Bliss: for babies born premature or sick, London, UK; 3 Division of Graduate Entry Medicine, School of Medicine, University of Nottingham, Nottingham, UK; 4 Division of Neonatal-Perinatal Medicine, Faculty of Medicine, Dalhousie University, IWK Health Centre, Nova Scotia, Canada

**Keywords:** hypoxic ischaemic encephalopathy, parenteral nutrition, breastfeeding, nnrd

## Abstract

**Introduction:**

Therapeutic hypothermia is standard of care for infants born ≥36 weeks gestation with hypoxic ischaemic encephalopathy (HIE); consensus on optimum nutrition during therapeutic hypothermia is lacking. This results in variation in enteral feeding and parenteral nutrition (PN) for these infants. In this study, we aim to determine the optimum enteral nutrition and PN strategy for newborns with HIE during therapeutic hypothermia.

**Methods and analysis:**

We will undertake a retrospective cohort study using routinely recorded electronic patient data held on the United Kingdom (UK) National Neonatal Research Database (NNRD). We will extract data from infants born ≥36 weeks gestational age between 1 January 2008 and 31 December 2016, who received therapeutic hypothermia for at least 72 hours or died during therapeutic hypothermia, in neonatal units in England, Wales and Scotland. We will form matched groups in order to perform two comparisons examining: (1) the risk of NEC between infants enterally fed and infants not enterally fed, during therapeutic hypothermia; (2) the risk of late-onset blood stream infections between infants who received intravenous dextrose without any PN and infants who received PN, during therapeutic hypothermia. The following secondary outcomes will also be examined: survival, length of stay, breast feeding at discharge, hypoglycaemia, time to full enteral feeds and growth. Comparison groups will be matched on demographic, maternal, infant and organisational factors using propensity score matching.

**Ethics and dissemination:**

In this study, we will use deidentifed data held in the NNRD, an established national population database; parents can opt out of their baby’s data being held in the NNRD. This study holds study-specific Research Ethics Committee approval (East Midlands Leicester Central, 17/EM/0307). These results will help inform optimum nutritional management in infants with HIE receiving therapeutic hypothermia; results will be disseminated through conferences, scientific publications and parent-centred information produced in partnership with parents.

**Trial registration number:**

NCT03278847; pre-results, ISRCTN47404296; pre-results.

**Strengths and limitations of this study**

- This retrospective observational study will use national, routinely recorded, clinical data held in the National Neonatal Research Database (NNRD); the robustness of NNRD data have been previously demonstrated for research purposes.
- The major limitation of this study is the non-random assignment of the intervention, which can lead to confounding as the decision for the intervention is influenced by patient characteristics.
- The NNRD holds a comprehensive data set of infants and maternal variables which permits the use of propensity analysis; this approach seeks to mimic randomised allocation and compare outcomes between matched cohorts with similar background characteristics but different exposures.
- A further strength is that data held in the NNRD were recorded prospectively and prior to the occurrence of the adverse outcome, therefore not susceptible to recall bias.

## Background

In high-resource countries, therapeutic hypothermia is standard of care for infants born ≥36 weeks gestational age with hypoxic ischaemic encephalopathy (HIE).^1^ Although the process of ‘cooling’ itself is well defined, based on high-quality randomised controlled trials,^1 2^ there are few data to inform provision of nutrition to infants with HIE during and after therapeutic hypothermia. This leads to variation in the provision of both enteral nutrition and parenteral nutrition (PN); a recent UK survey of nutrition practices during therapeutic hypothermia reported that only 31% of responding units have feeding guidelines for this group, 59% of neonatal units routinely start enteral feeding, 29% routinely administer PN and 37% provide intravenous dextrose fluids without feeds or PN.^3^

In relation to the enteral component of nutrition, withholding enteral (milk) feeds during therapeutic hypothermia is practised in the belief that it may reduce the risk of developing necrotising enterocolitis (NEC), despite lack of evidence that such an approach is beneficial even among higher risk groups, such as preterm infants.^4^ Conversely, starting enteral feeds during hypothermia is applied as it may have benefits, such as improved feed tolerance, earlier establishment of full enteral feeds, earlier discharge home^5^ and improved parental bonding.

Until enteral feeds are established, commonly used options for parenteral support during therapeutic hypothermia include provision of intravenous dextrose, provision of PN and a combination of both intravenous dextrose and PN. Intravenous dextrose provides sufficient hydration and energy to prevent hypoglycaemia, but does not provide protein and fat necessary for tissue growth; it is unknown how a short period of undernutrition impacts growth or the secondary and tertiary recovery phases that follow brain injury.^3 6 7^ On the other hand, PN is nutritionally superior but may increase the risk of infection, as seen in other paediatric studies.^8^ Furthermore, PN is expensive^9^ and, if not beneficial, or indeed harmful, avoiding or reducing its use would reduce costs.

The National Institute for Health Research (NIHR) Health Technology Assessment programme in the UK commissioned this study to determine the optimum enteral nutrition and PN strategies for newborns with HIE during and after therapeutic hypothermia. As adverse outcomes such as NEC are relatively rare among term and near-term infants receiving therapeutic hypothermia, a randomised controlled trial (RCT) would be costly, burdensome and time-consuming. In this protocol, we describe an observational study that will apply propensity score matching to form groups for comparison with near-identical distributions of background variables and therefore minimise the influence of potential confounders. We will use an existing population-level data set, the National Neonatal Research Database (NNRD), and will apply methods previously published.^10^ We aim to determine the optimum enteral nutrition and PN strategy for newborns with HIE during and after therapeutic hypothermia.

## Methods

### Design

To answer the research question ‘what is the optimum enteral and PN strategy for newborns during and after therapeutic hypothermia’, we will perform a retrospective cohort study using existing data held in the NNRD. We will apply a statistical technique called potential outcomes framework to compare primary and secondary outcomes between subgroups of infants with similar background characteristics exposed to different PN and enteral feeding strategies.

Eligible babies will be assigned to both an enteral (enterally fed or not fed) comparison and a parenteral comparison (intravenous dextrose or PN) and we will conduct two primary evaluations:

1. We will compare the rate of NEC between infants that were enterally fed versus not enterally fed during therapeutic hypothermia in the first three postnatal days. Being enterally fed is defined as receiving milk feeds of any type (including expressed maternal breast milk, expressed donor breast milk and artificial formula), by any route of administration (including nasogastric tube, bottle and suckling at the breast) and in any quantity, for at least 1 day.
2. We will compare the rate of late-onset (after day 3) blood stream infections (BSIs) between infants who received only intravenous dextrose versus those who received any PN during therapeutic hypothermia in the first three postnatal days. Receipt of PN is defined as receiving of any type (including standard, pre-prepared bags of nutrition and individually tailored PN), by any route of administration (including peripheral intravenous cannula, percutaneous central venous catheter or umbilical venous catheter) and in any volumes, for at least 1 day. Intravenous dextrose will include different volumes and routes of administration.

### Data source

This is a retrospective cohort study using anonymised, routinely recorded clinical data held in the NNRD. The NNRD holds data from all infants admitted to National Health Service (NHS) neonatal units in England, Scotland and Wales (approximately 90 000 infants annually); all NHS neonatal units in England and Wales have been contributing data to the NNRD since 2012, all NHS neonatal units in Scotland have been contributing to the NNRD since 2015. Contributing neonatal units are known as the UK Neonatal Collaborative (UKNC). Data are extracted from point-of-care neonatal electronic health records completed by health professionals during routine clinical care. A defined data extract, the Neonatal Dataset of approximately 450 data items,^11^ is transmitted quarterly to the Neonatal Data Analysis Unit at Imperial College London and Chelsea and Westminster NHS Foundation Trust where patient episodes across different hospitals are linked and data are cleaned (queries about discrepancies and implausible data configurations are fed back to health professionals and rectified).^12^ Data items include demographic and admission items (eg, maternal conditions, gestation, birth weight), daily items (eg, respiratory support, feeding information), discharge items (eg, feeding and weight at discharge) and ad hoc items (entered if and when they occur, eg suspected infection, ultrasound scan findings, abdominal X-ray findings).

### Time period

Data will be extracted for infants born between 1 January 2008 and 31st December 2017 and admitted to neonatal units contributing to the NNRD.

### Study setting

Neonatal units in Wales, England and Scotland.

### Eligibility criteria

Eligible infants within the NNRD include those who:

1. Were born and admitted to a neonatal unit between 1 January 2008 and 31 December 2017
2. Received care at a participating neonatal unit in England, Scotland and Wales (part of UKNC and therefore contributing data to the NNRD)
3. Have a recorded gestational age of ≥36^+0^ weeks^+days^ at birth
4. Were recorded as receiving therapeutic hypothermia for 72 hours or died during therapeutic hypothermia

Infants with missing data for principal background and outcome variables will be excluded.

### Definitions

#### Primary outcomes

For the comparison between infants fed milk feeds during therapeutic hypothermia with infants that were not, the primary outcome will be NEC, defined according to the UKNC case definition of Battersby *et al*.^13^ This case definition uses clinical and X-ray findings recorded using an ad hoc form in the summary electronic patient record from which the NNRD is derived. As these data are not recorded completely for some infants in the NNRD (in particular before 2010), we will also use two sensitivity analyses, using the following definitions:

- Severe NEC, defined as NEC confirmed at surgery or postmortem, or where the cause of death was NEC for those who died without a postmortem performed.^14^ Similar methods to those in the UK Neonatal Collaborative NEC Study^13^ will be used to identify infants with severe NEC. These involve using daily, diagnostic, abdominal X-ray and procedural variables held on the NNRD, and verifying these patient-level data with study leads where required.
- NEC (pragmatic definition), defined as a recorded diagnosis of NEC in an infant that received at least five consecutive days of antibiotics while kept nil by mouth.

For the comparison between infants that received parenteral (intravenous) nutrition during hypothermia with infants that received only intravenous dextrose, the primary outcome will be late-onset BSI (>3 days after birth). This will be defined according to the Healthcare Quality Improvement Partnership (HQIP) National Neonatal Audit Programme (NNAP) case definition as pure growth of pathogen from blood *OR* either a pure growth of a skin commensal or a mixed growth with ≥3 clinical signs at the time of blood sampling, recorded >3 days after birth.^15^ This definition uses data that are recorded using an ad hoc form in the summary electronic patient record from which the NNRD is derived. Data completeness for this ad hoc form is variable over time and across neonatal units; hence, we will also use a more pragmatic definition for late-onset BSI in a sensitivity analysis:

Late-onset BSI (pragmatic): Five consecutive days of antibiotic treatment that commenced more than 3 days after birth.

Secondary outcomes for both comparisons will include:

1. Survival, defined as alive at final neonatal unit discharge
2. Length of stay, defined as number of days between first neonatal unit admission and final neonatal unit discharge for surviving infants
3. Hypoglycaemia, defined as any diagnosis of hypoglycaemia recorded after therapeutic hypothermia is commenced and before the final neonatal unit discharge
4. Breast feeding at discharge, defined as any breast feeding (suckling at the breast) at discharge
5. Onset of breast feeding, defined as the first day at which an infant is recorded to be feeding at the breast (suckling; this does not include maternal breast milk given by bottle or nasogastric tube)
6. Time to first maternal breast milk feed (days), defined as the first day when an infant is recorded to be receiving maternal breast milk by any route (including suckling at the breast, by bottle or nasogastric tube)
7. Central venous line days, defined as number of recorded days an infant has a central venous line in situ
8. Growth, defined as standard deviation score (SDS) of the weight for postmenstrual age SDS at final neonatal unit discharge.

The following secondary outcome will be examined for the enteral comparison only:

1. Duration of PN, defined as the number of days that an infant is recorded to be receiving PN

### Statistical analysis

To address potential confounders (e.g., infants with hypotension who receive inotropes may be more likely to have feeds withheld and also to have poorer outcomes), we will use propensity matching to form subgroups of infants with similar characteristics including how sick they were when therapeutic hypothermia was started. As a consequence of such matching (balancing), any difference found is either a result of chance or reflects the difference in nutritional practice between the groups. For each infant, the propensity of the nutritional exposure (treatment) will be estimated by logistic regression that includes all background variables as covariates. Exposures that occur after therapeutic hypothermia is commenced will not be included in the propensity analysis as they are not background variables. The fitted propensities for the infants will be divided into 10 propensity groups (separated by deciles), or 20 propensity groups, depending on the extent to which the propensities of the two exposure groups overlap. Matched pairs will be formed within these groups with one infant from either exposure group. The pairs will be matched also on two important variables: (1) Cord blood gas pH (in bands: >7.0, 6.9–7.0, <6.9) and (2) birth year (in 2 year bands). The propensity model will be supplemented with transformations and interactions to obtain a good balance on all the background variables within matched groups. Balance plots will be used to assess the balance, and the procedure will be iterated until no further improvements can be achieved. Outcomes in the resulting two matched subgroups will then be compared using methods identical to those for a randomised trial with absolute and relative risks of serious adverse outcomes derived. The standard error of the estimate of the treatment effect will be obtained by combining the within and between-replication standard errors.^16^ All p values reported will be two-sided. Analyses will be performed using SAS V.9.3 (SAS Institute Inc, Cary, North Carolina, USA) and R.^17^

### Background covariates

The propensity model will include the following variables: demographic data items (gestational age at birth in weeks, birth weight SDS, sex, multiplicity); maternal factors (age, duration of rupture of membranes, fever, suspected chorioamnionitis, smoking status, ethnicity; maternal deprivation score based on lower layer super output area, hypothyroidism, diabetes, mode of delivery of infant, parity); infant factors at birth (Apgar score at 1 min and 5 min, chest compressions administered during resuscitation, emergency resuscitation drugs administered, intubated at resuscitation, umbilical cord base excess, time to first spontaneous breath); infant condition on admission prior to therapeutic hypothermia (admission mean blood pressure, glucose, heart rate, oxygen saturation, temperature); infant early-onset infection (positive blood or cerebrospinal fluid culture with a recognised pathogen recorded in the first 3 days); treatment on day 1 (infusion of inotropes, mechanical ventilation and treatment with inhaled nitric oxide); and organisational factors (required acute postnatal transfer within 24 hours, neonatal network).

Further details of all data items can be found in online supplemental data 1.

10.1136/bmjopen-2018-026739.supp1 Supplementary data

### Missing data

We will use imputation to address missing values, single imputation if a negligible fraction of data is missing, and multiple imputations otherwise. A variable will be dropped from the analysis if its value is missing (cannot be established) for a majority of the infants.

### Subgroup and sensitivity analyses

In addition to the sensitivity analyses that use alternate definitions for the primary outcomes already outlined, the following sensitivity analyses will be undertaken:

1. Restrict to data to 2012–2017 in England and Wales (where neonatal unit coverage by the NNRD was complete), to determine whether higher dropout rate seen prior to 2012 introduces bias that alters the direction or magnitude of findings
2. Restrict the population to infants for whom all enteral or parenteral feeding data have been actively recorded, that is, restrict the population to infants for whom enteral feeding, PN, intravenous glucose or nil by mouth have been recorded (and exclude infants for whom no data were entered during the first 4 days).
3. Excluding infants with extremely high and low propensity scores

The following subgroup analyses will be undertaken:

1. Excluding all infants whose first admission to neonatal care is from a postnatal ward; this will exclude cases for whom therapeutic hypothermia was administered following postnatal collapse

### Study sample size and power

It is estimated that approximately 7200 infants will meet the study inclusion criteria. Pilot data extracted from the NNRD show that in 2015, 809 infants met the study inclusion criteria, of which 38% (301/809) received enteral feeds and 29% (238/809) received PN during hypothermia. Using these proportions, a sample size of 7200 infants receiving therapeutic hypothermia will be able to detect (two-sided significance 5%, power 90%) a difference of 0.7% in NEC with 2000 matched pairs, assuming that the rate of NEC is negligible in the reference treatment, and 2% BSI with 1500 pairs, assuming rates of 1% and 3%.

### Steering committee

An independent Study Steering Committee appointed by the study funder (NIHR) will oversee the project.

### Parent, patient and public involvement

This study was commissioned by the NIHR. This study addresses two treatment uncertainties identified as research priorities by parents, patients and health professionals as part of the James Lind Alliance preterm birth priority setting partnership: prevention of NEC and prevention of infection. An author of this protocol and study coinvestigator (ES) is a parent of a baby who received therapeutic hypothermia in the newborn period; a further author of this protocol and study coinvestigator (MP) represents Bliss, the charity for babies born premature and sick. ES and Bliss have been involved in planning and designing the study, specifically in selection of study outcomes. In addition to academic dissemination, parent-centred materials will be developed in partnership with parent representatives to publicise the study results through conventional and social media channels.

### Ethics and dissemination

No patient identifiable information will be used in this study and only existing anonymised data held in the NNRD will be used. The Neonatal Data Analysis Unit (NDAU) holds UK Research Ethics Committee approval, 16/LO/1093, and Confidential Advisory Group approval, ECC 8-05(f/2010), to form the NNRD. Study-specific Research Ethics Committee approval was granted on 7 August 2017 by East Midlands—Leicester Central Research Ethics Committee (17/EM/0307). Health Research Authority approval was granted on 9 August 2017. Approval for this study was granted by the NDAU director and board at the steering group meeting on 11 October 2016. Approval from all neonatal units that make up the UKNC was granted on 1 September 2017. The study will be conducted in accordance with the recommendations for physicians involved in research on human subjects adopted by the 18th World Medical Assembly, Helsinki 1964 and later revisions.

Results will be presented at national and international academic conferences and published in peer-reviewed scientific publications. ES and MP will work (with the neonatal charity Bliss) to produce parent-centred information for dissemination through social media, online and to be distributed on neonatal units.

## Discussion

The optimum nutritional strategy for infants with HIE during therapeutic hypothermia is unclear. The findings of this study will help establish the risk–benefits of feeding versus withholding feeds and administering intravenous dextrose versus PN, on key neonatal morbidities, NEC and BSIs and other important secondary outcomes. The application of a novel analytical method (propensity scoring) to a population-level database (the NNRD) is well suited to studying important clinical uncertainties such as modes of nutrition, particularly in this situation where a randomised controlled trial (RCT) would be challenging due to high costs and the requirement for large sample sizes. The increasing availability of high-quality, routinely recorded health data, such as that held in the NNRD, has great potential to reduce clinical uncertainty and variation in neonatal care. As with all research, there are both avoidable and inherent biases in observational studies using routinely recorded health data. One well-identified potential bias stems from incomplete or unclear reporting^20^; by publishing our study protocol a priori, in accordance with the REporting of studies Conducted using Observational Routinely-collected Data (RECORD) guidance,^21^ and including details of the database and data items to be used and describing the matching process with the covariates to be used, we hope to reduce avoidable bias in this study.

The proposed study has a number of strengths. The robustness of core NNRD data (birth weight, sex, length of stay and death) has been previously demonstrated for research purposes,^14 22^ and data are used for multiple purposes including national audit including the UK Healthcare Quality Improvement PartnershipHQIP-funded NNAP and analyses for the Department of Health, NHS England and the Chief Medical Officer. Routine data have the advantage of being recorded prospectively prior to the occurrence of the adverse outcome, therefore not susceptible to recall bias. In contrast to RCTs, studies that use population-based data sources, such as in this study, avoid recruitment bias: data from all eligible infants are included and hence findings are more generalisable. The major limitation of observational studies is the non-random assignment of the intervention, which can lead to confounding as the decision for the intervention is influenced by patient characteristics. However, a major strength of the NNRD is its comprehensive data set of infants and maternal variables; this permits the use of propensity analysis, which seeks to mimic randomised allocation and compare outcomes between matched cohorts with similar background characteristics but different exposures.

At present, there is insufficient evidence to recommend optimal feeding and PN strategies during therapeutic hypothermia for infants with HIE. This protocol describes a retrospective observational study that seeks to address this important clinical uncertainty.

## Supplementary Material

Reviewer comments
