# Supplementary material for: Study protocol: optimising newborn nutrition during and after neonatal therapeutic hypothermia in the United Kingdom: observational study of routinely collected data using propensity matching
Source: BMJ Open. 2018 Oct 23;8(10):e026739. doi: 10.1136/bmjopen-2018-026739 (PMC6224768; doi:10.1136/bmjopen-2018-026739)
Supplement: Supplementary data [file bmjopen-2018-026739supp001.pdf]

Data fields for extraction: Optimising newborn nutrition during therapeutic hypothermia: an observational study using routinely collected data

Population

- Babies admitted to a NHS neonatal unit in England, Scotland and Wales
- Gestational age at birth 36+0 (weeks+days) or greater
- Received therapeutic hypothermia for at least 3 days, or died on a day where they were recorded as receiving therapeutic hypothermia

  The following recorded in *daily care neurology* field: Therapeutic hypothermia induced - for 3 or more consecutive days

- Admitted to neonatal unit January 1st 2008 to December 31st 2017

Data source

- Data will be extracted from the National Neonatal Research Database (NNRD) at the Neonatal Data Analysis Unit (NDAU) at Imperial College London.
- The NNRD contains a predefined set of variables (the Neonatal Data Set, an authorised NHS Information Standard) extracted at regular intervals from the Electronic Patient Record of every admission to a NHS neonatal unit in England, Wales, and Scotland, cleaned and merged across multiple patient episodes, to create a single data file for each patient.

**Treatment**	
**Variable**	**Data items**
Enteral Nutrition	**ENTERAL FEEDING GROUP DEFINED AS**<br>Any of the following items entered in the *Daily Care Fluids and Feeding* during first 3 days<br>• Any entry (1-6) under ENTERAL FEED TYPE GIVEN<br>• OR any entry (0-88) under FORMULA MILK OR MILK FORTIFIER TYPE<br>• OR any value >0 for TOTAL VOLUME OF MILK RECEIVED<br>• OR any entry (1-8) under ENTERAL FEEDING METHOD<br><br>**NON-ENTERAL FEEDING GROUP DEFINED AS**<br>All other babies not fulfilling above criteria
Parenteral Nutrition	**PARENTERAL NUTRITION GROUP DEFINED AS**<br>Any of the following items entered in the *Daily Care Fluids and Feeding* during first 3 days<br>• Y entry for PARENTERAL NUTRITION RECEIVED INDICATOR<br>OR

	The following drug code entered in the *Daily care medication* during first 3 days<br>• 1010238 *Total parenteral nutrition*<br><br>**NON- PARENTERAL NUTRITION GROUP DEFINED AS**<br>All other babies not fulfilling above criteria<br><br>For sensitivity analyses also extract<br>• *Daily Care Fluids and Feeding* INTRAVENOUS INFUSION OF GLUCOSE AND ELECTROLYTE SOLUTION RECEIVED INDICATOR = Y/N

**Outcome**	
**Variable**	**Data items**
Severe NEC	Gestational age specific NEC score based on Battersby et al., JAMA Pediatrics 2017. Data items needed:<br>ABDOMINAL X-RAYS (EPISODIC)<br>• CONDITION SEEN IN ABDOMEN DURING X-RAY (NNRD field ID: XRayAppearances)<br>• ABDOMINAL X-RAY PERFORMED REASON (NNRD field ID: ClinicalFindings)<br>• TRANSFERRED FROM NEONATAL INTENSIVE CARE UNIT FOR NECROTISING ENTEROCOLITIS MANAGEMENT INDICATOR (NNRD field ID: TransferredForFurtheManagement)<br>• LAPAROTOMY FOR NECROTISING ENTEROCOLITIS INDICATION CODE<br>• VISUAL INSPECTION CONFIRMED NECROTISING ENTEROCOLITIS DURING LAPAROTOMY INDICATOR<br>• HISTOLOGY CONFIRMED NECROTISING ENTEROCOLITIS FOLLOWING LAPAROTOMY INDICATOR<br>• POSTMORTEM CONFIRMED NEC<br>• CAUSE OF DEATH<br>Only available following introduction of ABDOMINAL X-RAY (EPISODIC) field
Necrotising enterocolitis (non-UKNC definition)	The following entered in the *Daily Care Gastrointestinal* at any point during neonatal unit stay<br>• Any entry (1 or 2) for TREATMENT TYPE FOR NECROTISING ENTEROCOLITIS<br>OR the following diagnostic codes<br>• 1010683 *Necrotising enterocolitis – suspected*<br>• 10708 *Necrotising enterocolitis – Perforated*<br>• 15809 *Necrotizing enterocolitis*<br><br>AND<br>5 or more days nil by mouth

	Defined by the *Daily Care Fluids and Feeding* for a continuous period of 5 days<br>• No under ENTERAL FEED TYPE GIVEN<br>• No entry under FORMULA MILK OR MILK FORTIFIER TYPE<br>• No value OR 0 for TOTAL VOLUME OF MILK RECEIVED<br>• No entry under ENTERAL FEEDING METHOD<br><br>WHILE ALSO RECEIVING<br>5 or more days of antibiotics over the same 5 days as the baby was nil by mouth, defined as 5 consecutive days of any of the following *Daily care medication*<br>• 1010155 Benzyl Penicillin<br>• 1010158 Augmentin<br>• 1010179 Flucloxicillin<br>• 500012 Flucloxacillin<br>• 500016 Gentamicin<br>• 500072 *Co-amoxiclav*<br>• 500086 Co-amoxiclav<br>• 500084 Ciprofloxacin<br>• 500029 Netilmicin<br>• 500002 Amikacin<br>• 500211 Tazocin<br>• 500023 Metronidazole<br>• 500040 Vancomycin<br>• 500007 Cefotaxime<br>• 500004 Ampicillin<br>• 500009 Cefuroxime<br>• 500008 Ceftazidime<br>• 500175 Ceftriaxone<br>• 500032 Piperacillin<br>• 500206 *Oflacillin*<br>• 500005 Azlocillin<br>• 1010171 Linezolid<br>• 1010271 *Cefalexin*<br>• 1010139 Amoxicillin<br>• 500070 Amoxicillin<br>• 500128 Meropenem<br>• 500118 Imepenem<br>• 500145 *Imipenem*<br>• 500069 Ambisome (Liposomal Amphotericin)<br>• 500003 Amphotericin<br>• 1010195 Amphotericin Liposomal
Late onset blood stream infection NNAP definition	**NNAP definition**<br>Defined from *Infection Cultures (Episodic)* recorded after day 3<br>• Pure growth of pathogen from blood<br>OR<br>• Pure growth of pathogen from CSF<br>OR

	• Either a pure growth of a skin commensal or a mixed growth with ≥3 clinical signs at the time of blood sampling
Late onset infection, non-NNAP	5 consecutive days of antibiotic treatment defined as 5 consecutive days of any of the following (including in combination and changing during the 5 days) after day 3<br>*Daily care medication*<br>• 1010155 Benzyl Penicillin<br>• 1010158 Augmentin<br>• 1010179 Flucloxicillin<br>• 500012 Flucloxacillin<br>• 500016 Gentamicin<br>• 500072 *Co-amoxiclav*<br>• 500086 Co-amoxiclav<br>• 500084 Ciprofloxacin<br>• 500029 Netilmicin<br>• 500002 Amikacin<br>• 500211 Tazocin<br>• 500023 Metronidazole<br>• 500040 Vancomycin<br>• 500007 Cefotaxime<br>• 500004 Ampicillin<br>• 500009 Cefuroxime<br>• 500008 Ceftazidime<br>• 500175 Ceftriaxone<br>• 500032 Piperacillin<br>• 500206 *Oflacillin*<br>• 500005 Azlocillin<br>• 1010171 Linezolid<br>• 1010271 *Cefalexin*<br>• 1010139 Amoxicillin<br>• 500070 Amoxicillin<br>• 500128 Meropenem<br>• 500118 Imepenem<br>• 500145 *Imipenem*<br>• 500069 Ambisome (Liposomal Amphotericin)<br>• 500003 Amphotericin<br>• 1010195 Amphotericin Liposomal
Survival to discharge	Defined from the *Discharge Details* from final neonatal unit stay<br>• DISCHARGE DESTINATION FROM NEONATAL CRITICAL CARE = 1, 2, 4, 5, 6 (NOT code 3, *Died*)
Length of neonatal unit stay	Defined as the total number of days a baby received neonatal care (any level of care) from *Daily Care General Information* - LOCATIONS OF HIGHEST LEVEL OF CARE
Hypoglycaemia	Defined as any of the following *diagnostic codes* recorded at any time during an infants neonatal units stay:<br>• 15771 Iatrogenic neonatal hypoglycaemia<br>• 15773 Neonatal hypoglycaemia

Breastfeeding at discharge	Defined from final day of neonatal care entry in *Daily Care Fluids and Feeding* of<br>• ENTERAL FEED TYPE GIVEN = code 1 (Breastfeeding)<br>OR<br>• ENTERAL FEEDING METHOD = code 1 (breast)<br>Where final day is not entered, penultimate day will be used
Onset of breastfeeding	Number of days until first entry in *Daily Care Fluids and Feeding* of<br>• ENTERAL FEED TYPE GIVEN = code 1 (Breastfeeding)<br>OR<br>• ENTERAL FEEDING METHOD = code 1 (breast)
Time to first maternal breast milk feed	First day where a baby is recorded to be receiving maternal breast milk by any route (including suckling at the breast, by bottle or nasogastric tube) defined as *Daily Care Fluids and Feeding* of<br>• ENTERAL FEED TYPE GIVEN = code 1 (Breastfeeding); 2 (Mothers fresh expressed breast milk); 3 (Mothers frozen expressed breast milk); 4 (Donor expressed breast milk)<br>OR<br>• ENTERAL FEEDING METHOD = code 1 (breast)
Duration of parenteral nutrition	Defined as the number of days UNTIL a baby has:<br>• *Daily Care Fluids and Feeding* PARENTERAL NUTRITION RECEIVED INDICATOR = N<br>AND<br>• The following drug code NOT entered in the *Daily care medication:* 1010238 *Total parenteral nutrition*<br><br>This analysis will only be performed for the ENTERAL COMPARISON
Number of days an infant has a central venous line in situ	Defined as the number of days that has a baby has:<br>• *Daily Care Fluids and Feeding* VASCULAR LINE TYPE IN SITU = code 3 (Umbilical venous line); 4 (Percutaneous central venous line ('long line'); 5 (Surgically inserted central venous line)
Weight SDS at discharge	Defined as the following data item on the final day of neonatal care:<br>• *Daily Care General Information* PERSON WEIGHT IN GRAMS<br>Where final day is not entered, penultimate day will be used

**Background variables for forced matching**	
**Variable**	**Data items**
Cord blood gas pH in bands	*Labour and Delivery Details* UMBILICAL CORD BLOOD PH LEVEL (ARTERIAL)<br>*Or if not recorded use*<br>*Labour and Delivery Details* UMBILICAL CORD BLOOD PH LEVEL (ARTERIAL)<br>Tricotomise into bands: >7.0, 6.9-7.0, <6.9

Birth year: in 2 year bands	*Baby Demographics* YEAR AND MONTH OF BIRTH (BABY)<br>Continuous in 2 year bands

**Background variables for matching**	
**Variable**	**Data items**
Gestational age week	*Baby Demographics* GESTATION LENGTH (AT DELIVERY): Gestational weeks and days
Birthweight standard deviation score (SDS)	*Baby Demographics* BIRTH WEIGHT
Sex	*Baby Demographics* PERSON PHENOTYPIC SEX
Multiplicity	*Labour and Delivery Details* BIRTH ORDER (MATERNITY SERVICES)<br>*Labour and Delivery Details* NUMBER OF FETUSES (NOTED DURING PREGNANCY EPISODE)<br>Dichotomous 1/other number
Maternal age	*Parents Demographics* YEAR OF BIRTH (MOTHER)<br>(age continuous in years)
Maternal duration of rupture of membranes (time in hours)	*Labour and Delivery Details* RUPTURE OF MEMBRANES DATE TIME *or* RUPTURE OF MEMBRANES YEAR AND MONTH and *NUMBER OF MINUTES (BIRTH TO EVENT)*<br>(continuous)
Maternal pyrexia	*Labour and Delivery Details* SIGNIFICANT MATERNAL PYREXIA IN LABOUR INDICATOR<br>(Y/N)
Maternal suspected chorioamnionitis	*Labour and Delivery Details* INTRAPARTUM ANTIBIOTICS GIVEN INDICATORS
Maternal smoking status	*Pregnancy Details* MOTHER CURRENT SMOKER AT BOOKING INDICATOR<br>(categorical, codes 1-6)
Maternal ethnicity	*Parents Demographics* ETHNIC CATEGORY (MOTHER)<br>(categorical)
Maternal deprivation score (from lower super output area)	*Parents Demographics* POSTCODE OF USUAL ADDRESS (LSOA)
Maternal hypothyroid (Y/N)	*Pregnancy Details* MATERNITY COMPLICATING MEDICAL DIAGNOSIS TYPE<br>Dichotomous: Y=code 16 (endocrine disorder), N=any other or no code
Maternal diabetes (Y/N)	*Pregnancy Details* MATERNITY COMPLICATING MEDICAL DIAGNOSIS TYPE<br>Dichotomous: Y=code 08 (diabetes)

	OR<br>*Pregnancy Details* MATERNITY OBSTETRIC DIAGNOSIS TYPE<br>Dichotomous: Y=code 06 (gestational diabetes mellitus)<br>N=any other or no code
Mode of delivery of infant (vaginal or Caesarean)	*Labour and Delivery Details* MODE OF DELIVERY<br>Categorical: codes=1-4<br>AND<br>*Labour and Delivery Details IN LABOUR BEFORE CAESARIAN SECTION INDICATOR*=Y/N
Parity of mother (primiparous Y/N)	*Pregnancy Details* PREGNANCY TOTAL PREVIOUS PREGNANCIES<br>Dichotomous: code 00=Y; code 01-29=N
Apgar score at 1 minute	*Labour and Delivery Details* APGAR SCORE (1 MINUTE)<br>Continuous: 0-10
Apgar score at 5 minutes	*Labour and Delivery Details* APGAR SCORE (5 MINUTE)<br>Continuous: 0-10
Chest compressions administered	*Labour and Delivery Details* NEONATAL RESUSCITATION METHOD<br>Dichotomous: Code 16=Y; any other code=N
Emergency resuscitation drugs administered	*Labour and Delivery Details* NEONATAL RESUSCITATION METHOD<br>Dichotomous:<br>Y= code 17 (Adrenaline) OR 88 (any other drug)<br>N= any other codes OR no code
Intubated at resuscitation	*Labour and Delivery Details* NEONATAL RESUSCITATION METHOD<br>Dichotomous: Code 15=Y; any other code=N
Umbilical cord base excess	*Labour and Delivery Details* UMBILICAL CORD BLOOD BASE EXCESS CONCENTRATION (ARTERIAL)<br>Continuous<br>OR if not available use<br>*Labour and Delivery Details* UMBILICAL CORD BLOOD BASE EXCESS CONCENTRATION (VENOUS)
Time to first spontaneous breath	*Labour and Delivery Details* TIME BETWEEN DELIVERY AND SPONTANEOUS RESPIRATION CODE<br>Continuous
Admission mean blood pressure	*Admission Details* MEAN ARTERIAL BLOOD PRESSURE (ON ADMISSION TO NEONATAL CRITICAL CARE)<br>Continuous
Admission blood glucose	*Admission Details* BLOOD GLUCOSE CONCENTRATION (ON ADMISSION TO NEONATAL CRITICAL CARE)<br>Continuous
Admission heart rate	*Admission Details* HEART RATE (ON ADMISSION TO NEONATAL CRITICAL CARE)<br>Continuous
Admission oxygen saturation	*Admission Details* OXYGEN SATURATION (ON ADMISSION TO NEONATAL CRITICAL CARE)<br>Continuous
Admission temperature	*Admission Details* TEMPERATURE (ON ADMISSION TO NEONATAL CRITICAL CARE)<br>Continuous

Positive blood or cerebrospinal fluid culture with a recognised pathogen recorded in the first 3 days	Defined from *Infection Cultures (Episodic)* recorded up to and including day 3<br>• Pure growth of pathogen from blood<br>OR<br>• Pure growth of pathogen from CSF
Treatment for low blood pressure with an intravenous inotrope (e.g. dopamine, noradrenaline)	*Daily Care Medication* on day 1 only<br>• 500098 Dopamine<br>• 500096 Dobutamine<br>• 500056 Adrenaline<br>• 500210 Noradrenaline<br>• 500116 Hydrocortisone<br>• 1010173 Milrinone<br>Dichotomous: any of above=Y, none of above=N<br>OR<br>*Daily Care Cardiovascular* INOTROPE INFUSION RECEIVED INDICATOR Y/N
Mechanical ventilation method	*Daily Care Respiratory* on day 1 only; RESPIRATORY SUPPORT MODE<br>Dichotomous: Codes 1, 2, 3=Y; any other or no code =N
Received inhaled nitric oxide (Y/N)	*Daily Care Respiratory* on day 1 only; NITRIC OXIDE GIVEN INDICATOR<br>Dichotomous: Y/N
Required acute postnatal transfer, within 24 hours (Y/N)	*Admission Details* SITE CODE (OF ADMITTING NEONATAL UNIT) or ORGANISATION CODE (OF ADMITTING NEONATAL UNIT)<br>Different from<br>*Baby Demographics* SITE CODE (OF ACTUAL PLACE OF DELIVERY) or ORGANISATION CODE (OF ACTUAL PLACE OF DELIVERY)<br>And<br>*Baby Demographics* EPISODE NUMBER
Neonatal network	*Baby Demographics* SITE CODE (OF ACTUAL PLACE OF DELIVERY) or ORGANISATION CODE (OF ACTUAL PLACE OF DELIVERY)

**Other descriptive variables**	
**Variable**	**Data items**
Birth head circumference	*Baby Demographics* BIRTH HEAD CIRCUMFERENCE
Birth length	*Baby Demographics* BIRTH LENGTH
Worse Base deficit	*Baby Demographics* BASE DEFICIT CONCENTRATION (WORST WITHIN 12 HOURS AFTER BIRTH)
Place of booking	*Pregnancy Details* MOTHER ANTENATALLY BOOKED INDICATOR
Maternal obstetric diagnosis	*Pregnancy Details* MATERNITY OBSTETRIC DIAGNOSIS TYPE (CURRENT PREGNANCY)

Maternal medical diagnosis (current pregnancy)	*Pregnancy Details* MATERNITY MEDICAL DIAGNOSIS TYPE (CURRENT PREGNANCY)
Cigarettes per day	*Pregnancy Details* CIGARETTES PER DAY (MOTHER AT BOOKING)
Onset of labour	*Labour and Delivery Details* LABOUR OR DELIVERY ONSET METHOD CODE
Instrument of delivery	*Labour and Delivery Details* DELIVERY INSTRUMENT TYPE
Presentation at delivery	*Labour and Delivery Details* PRESENTATION AT DELIVERY
In labour?	*Labour and Delivery Details* IN LABOUR BEFORE CAESARIAN SECTION INDICATOR
Time to admission	*Admission Details* CRITICAL CARE START YEAR AND MONTH and NUMBER OF MINUTES (BIRTH TO EVENT)
Level of neonatal care	*Level of care for each day*
Death details	*Cause of death* DEATH CAUSE ICD CODE (DURING NEONATAL CRITICAL CARE PERIOD)
Blood transfusion	*Daily care blood transfusion* BLOOD TRANSFUSION PRODUCT TYPE
Maternal occupation	*Parents Demographics (withheld)* OCCUPATION MOTHER (SNOMED CT)
