# Supplementary material for: Study protocol: optimising newborn nutrition during and after neonatal therapeutic hypothermia in the United Kingdom: observational study of routinely collected data using propensity matching
Source: BMJ Open. 2018 Oct 23;8(10):e026739. doi: 10.1136/bmjopen-2018-026739 (PMC6224768; doi:10.1136/bmjopen-2018-026739)
Supplement: Reviewer comments [file bmjopen-2018-026739.reviewer_comments.pdf]

PEER REVIEW HISTORY

BMJ Open publishes all reviews undertaken for accepted manuscripts. Reviewers are asked to complete a checklist review form (**http://bmjopen.bmj.com/site/about/resources/checklist.pdf**) and are provided with free text boxes to elaborate on their assessment. These free text comments are reproduced below.

ARTICLE DETAILS

**TITLE (PROVISIONAL)**	Study Protocol: Optimising newborn nutrition during and after neonatal therapeutic hypothermia in the United Kingdom: Observational study of routinely collected data using propensity matching
**AUTHORS**	Battersby, Cheryl; Longford, Nick; Patel, Mehali; Selby, Ella; Ojha, Shalini; Dorling, Jon; Gale, Chris

This article was not externally reviewed at BMJ Open. Protocols that have been independently assessed prior to submission to BMJ Open are usually fast-tracked to publication on the grounds that further substantial changes will not be possible. This independent assessment will usually be external, independent review for both a substantial grant award from a non-commercial or government funder and ethics approval.
